# SARS-CoV-2 transmission during an indoor professional sporting event

**DOI:** 10.1038/s41598-021-99997-0

**Published:** 2021-10-20

**Authors:** Johannes Pauser, Chantal Schwarz, James Morgan, Jonathan Jantsch, Matthias Brem

**Affiliations:** 1 Curathleticum Nuemberg, Nuremberg, Germany; 2 Institute of Clinical Microbiology and Hygiene, University Hospital of Regensburg and University of Regensburg, Regensburg, Germany; 3 Paracelsus Medical University, Nuremberg, Germany; 4 Department of Orthopedic and Trauma Surgery, External Faculty Member, Friedrich-Alexander University Erlangen-Nuremberg, Nuremberg, Germany

**Keywords:** SARS-CoV-2, SARS virus, Viral epidemiology, Viral transmission

## Abstract

Sporting events with spectators can present a risk during the COVID-19 pandemic of becoming potential superspreader events that can result in mass-infection amongst participants—both sportspeople and spectators alike. In order to prevent disease transmission, many professional sporting bodies have implemented detailed hygiene regulations. This report analyzes SARS-CoV-2 transmission during a professional sports event (2nd division professional basketball in Germany). Whilst social distancing in this context is not always possible, the rate of infection was significantly reduced by wearing face masks that cover the mouth and nose. There was no infection amongst individuals who continuously wore medical particle filter masks (Category KN95/FFP2 or higher) during this sporting event.

## Introduction

Sporting events could represent so-called “superspreader events” and could be instrumental in fueling the current COVID-19 pandemic^1^. Therefore, soon after the emergence of SARS-CoV-2 pandemic, public and private team sport events as we knew them were prohibited in order to curtail further dissemination of SARS-CoV-2. Numerous sports associations, such as the German Football Association (DFB) and the Deutsche Fußballliga (DFL)^2^ as well as the Basketball Bundesliga (German Basketball League)^3^, have issued hygiene guidelines and excluded the public from these events. These measures allowed professional sports to continue to be played.

SARS-CoV-2 can be transmitted via contact and droplets^4,5^. In addition, the airborne route of transmission of SARS-CoV-2 is now appreciated to play an key role in the spread of SARS-CoV-2^5–8^. In contrast to outdoor sports events^9^, indoor sports, however, could play a very significant risk for the spread of SARS-CoV-2, especially if ventilation is inadequate. For instance, a recent study demonstrates that exchange rates and air flow within an indoor environment play an important role in SARS-CoV-2 transmission^5^.

Intensive, physically demanding sport such as professional basketball does not allow the continuous use of face masks. In contrast to players actively engaged in the game, individuals not directly participating in the sporting activity are able to wear face masks.

Here, we report on a SARS-CoV-2 spreading event during a professional indoor sport activity. In this setting, we assessed the contribution of face masks in preventing SARS-CoV-2 transmission retrospectively.

## Methods

This study was approved by the Regensburg University Ethics Committee (20-2144-101). In this project, we retrospectively analyzed an outbreak that occurred during a professional sport event retrospectively. All players, coaches and other persons present at the sporting event were approached and asked whether they are willing to provide the data necessary for the evaluation. The event took place in a sports facility that could potentially accommodate up to 1540 spectators. Data on exchange rates of air and air flow were not available. Excluding the athletes on the playing field, all other people present at the event were asked to stay at least 1.5 m apart from other people. In total 69 persons were present at the event (21 players and 48 staff/ assistants). 88% (61 out of 69) gave their written consent to participate in the study. All research was performed in accordance with relevant national regulations and in accordance with the Declaration of Helsinki. Informed consent was obtained from all participants. Pseudoanonymization of participants was carried out and the participants of the study were questioned with regard to age, gender, previous illnesses, protective measures taken, exposure time, clinical symptoms and data, as well as laboratory parameters (where available), and the course of the disease. Statistical analysis was performed using GraphPad Prism v6.

## Results

Prior to a sporting event taking place without spectators in November 2020 a risk assessment of contact to SARS-CoV-2 and an evaluation of symptoms amongst participants was carried out—conforming with the standard hygiene concept implemented at that time by the 2nd division of the German professional club basketball (2. Basketball Bundesliga) that did not include PCR-testing to detect SARS-CoV-2 amongst asymptomatic individuals^10^. Immediately prior to the beginning of the sport event, all participants were asked about symptoms and temperature was measured. No participant had a raised temperature or reported symptoms associated with COVID-19. The indoor hall was empty prior to the start of the sport event.

After the event, a player of team B reported that he developed nonspecific symptoms of a respiratory tract infection. This participant tested positive for SARS-CoV-2. Subsequently, respiratory PCR testing for SARS-CoV-2 was performed in approximately 90% of the participants. SARS-CoV-2 was detected in 65% of the tested participants at a median of 4.00 days (interquartile range: 3.00–6.75 days) after the sporting event. Positively tested participants developed symptoms 4.00 days (median, interquartile range: 3.00–5.00 days) after the sporting event. There was no significant difference between both participating teams and other participants respectively concerning the commencement of symptoms (Table 1). Three participants suffered a severe infection and were hospitalized.

**Table 1:** Appearance of COVID-19-symptoms after the sport event.

Group	Median (in days)	Interquartile range (in days)
Team A	3.00	2.00–4.00
Team B	2.00	0.25–4.50
Other participants	4.00	3.00–6.00

A Kruskal–Wallis-Test did not indicate any significant difference between the groups.

**Figure 1:**
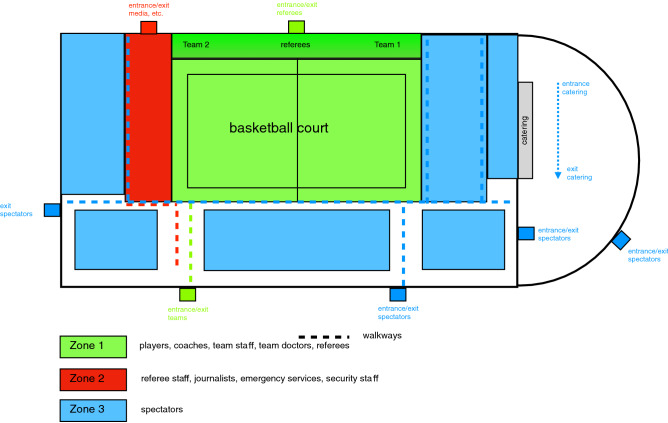
Sports facility and its zones according to the health and safety/hygiene concept implemented by the 2. Basketball Bundesliga (not true to scale).

The health and safety/hygiene concept implemented by the 2. Basketball Bundesliga involves dividing the basketball stadium into three zones^10^. Zone 1 is the basketball court including a three-metre security area surrounding the entire court. The players, coaches, team staff, team doctors, and referees are allowed in this area. Zone 2 is designated for referee staff, journalists, emergency services and security staff whilst Zone 3 is intended for spectators (Fig. 1).

26 out of the 36 SARS-CoV-2 positive participants were in zone 1 where the game took place. 3 of the 26 SARS-CoV2-infected participants in zone 1 wore masks continuously. Amongst those who did not wear a medical face mask during the entire event and did not become infected despite being in Zone 1 was a participant who had already previously suffered a documented SARS-CoV-2 infection, and therefore could be assumed to have immunity to the disease. In comparison, wearing a face mask (medical face mask (community masks and/or surgical masks) or particle filter masks (FFP2, FFP3 or KN95)) reduced the risk of SARS-CoV-2-transmission from 83 to 46% amongst all participants who were tested for SARS-CoV-2. The difference in the proportion of individuals wearing a face mask and participants not using a face mask who were not tested SARS-CoV-2 positive is very significant (Table 2, two-tailed Fisher’s exact test, p = 0.0055). Of utmost importance, our data also indicate that wearing masks does not provide 100% protection. However, four individuals who continuously wore a medical particle filter mask (category KN95, FFP2 or FFP3) throughout the game showed no evidence of infection despite these participants being in Zone 1 during the sporting event. In the group of participants who wore community and/or surgical masks physical distancing did not result in any further reduction in the rate of disease transmission. Transmission occurred amongst 60% of participants wearing community and/or surgical face masks in Zone 1, 55% in Zone 2, and 50% in Zone 3 (Table 3).

**Table 2:** Contingency table on the impact of masks^a^ on SARS-CoV-2 transmission.

Group	Neg SARS-CoV-2	Pos SARS-CoV-2
Mask	14	12
No mask	5	24

^a^Medical face masks (community masks and/or surgical masks) or particle filter masks (FFP2, FFP3, KN95).

**Table 3:** Impact of physical distancing on SARS-CoV-2 transmission in the group of participants using medical face masks (community masks and/or surgical masks).

Group	Pos SARS-CoV-2	Neg SARS-CoV-2
Zone 1	3 (60%)	2 (40%)
Zone 2	6 (55%)	5 (45%)
Zone 3	3 (50%)	3 (50%)

## Discussion

This report shows that transmission of SARS-CoV-2 can occur during indoor sporting events despite the implementation of a hygiene concept, in particular in the absence of PCR testing for SARS-CoV-2 for all asymptomatic participants prior to events taking place. There was no in-depth laboratory analysis of SARS-CoV-2 in November 2020 (with respect to the precise SARS-CoV-2 virus type involved) during which time this event took place. At the time this report was written, there were no further laboratory samples available from the respective infections suffered at this event, therefore a further retrospective molecular analysis was not possible.

Our data clearly support the notion that wearing facial masks reduced the rate of infection^11^. Surprisingly, in our retrospective study we did not detect a difference in transmission in persons that were allocated to stay in zone 1, 2 or 3. However, since contact time and time spent in the respective zone was not monitored continuously this does not allow to conclude that distancing is useless. There is strong evidence that distancing helps to contain spreading of SARS-CoV-2^12–14^. Of utmost interest, however, participants who continuously wore a particle filter masks (category KN95/ FFP2 or higher) were fully protected. This strongly suggests that appropriate protective equipment is able to prevent SARS-CoV-2 transmission and that particle filter masks are especially useful in this regard.

As it is not possible to wear medical face masks whilst undertaking an intensive, physically demanding sport such as professional basketball, it is likely that infection resulted from airborne transmission of SARS-CoV-2 and played a major role in participants contracting SARS-CoV-2 within the basketball stadium during the match. These findings correspond with other reports which have observed the transmission of SARS-CoV-2 during rigorous physical activity^15–17^ in particular indoor activities^18,19^. Interestingly, a recent study carried out in the sport of rugby—which is not played indoors—has shown that there appears to be no risk of SARS-CoV-2 transmission^20^. Nonetheless, experience gained during the UEFA EURO 2020 event clearly shows that the behavior of spectators can critically contribute to SARS-CoV-2 transmission rates during outdoor sport events in general, especially in scenarios where many spectators are present^12^. Overall, the infection risks of carrying out sporting events indoors and outdoors with respect to transmission of SARS-CoV-2 have not been finally established yet. However, it appears likely that the risk of SARS-CoV-2 transmission in an indoor sporting environment is higher.

Since asymptomatic people substantially contribute to the spread of SARS-CoV-2^21^, testing of active participants prior to the sport event may be a suitable measure to reduce the risk of SARS-CoV-2 transmission. This testing strategy may also include vaccinated participants, since vaccination does not necessarily prevent infection and dissemination of SARS-CoV-2 infection under all circumstances^22,23^. Antigen tests seem not to be sufficient to identify all potentially SARS-CoV-2 positive sport participants^24^. Consequently, in the future it appears to be important to carry out PCR tests for detecting possible asymptomatic SARS-CoV-2 infections amongst actively participating players especially prior to indoor sporting events in order to prevent the mass transmission of this disease.

## References

[1] Sassano M, McKee M, Ricciardi W, Boccia S (2020). Transmission of SARS-CoV-2 and other infections at large sports gatherings: A surprising gap in our knowledge. Front. Med. (Lausanne).

[2] Deutscher Fussball-Bund and DFL. Task Force Sportmedizin/ Sonderspielbetrieb im Profifussball. Version 2. (2020).

[3] e@syCredit® BBL (Basketball Bundesliga). Konzept für den Sonderspielbetrieb zur Wiederaufnahme der Saison 2019/2020. Version 2.0. (2020).

[4] Schulze-Robbecke R, Reska M, Lemmen S (2020). Laryngorhinootologie.

[5] Moritz S (2021). The risk of indoor sports and culture events for the transmission of COVID-19. Nat. Commun..

[6] Greenhalgh T (2021). Ten scientific reasons in support of airborne transmission of SARS-CoV-2. Lancet.

[7] Leung NHL (2021). Transmissibility and transmission of respiratory viruses. Nat. Rev. Microbiol..

[8] Jarvis MC (2020). Aerosol transmission of SARS-CoV-2: Physical principles and implications. Front. Public Health.

[9] England R (2021). The potential for airborne transmission of SARS-CoV-2 in sport: A cricket case study. Int. J. Sports Med..

[10] Hygieneleitfaden BARMER 2. Basketball Bundesliga. (2020).

[11] Brooks JT, Butler JC (2021). Effectiveness of mask wearing to control community spread of SARS-CoV-2. JAMA.

[12] Smith, J. A. E. *et al.* Public health impact of mass sporting and cultural events in a rising COVID-19 prevalence in England. *Preprint* (2021). 10.1017/S0950268822000188 PMC9058658 35094727

[13] Auranen K (2021). Social distancing and SARS-CoV-2 transmission potential early in the epidemic in Finland. Epidemiology.

[14] Morley CP (2020). Social distancing metrics and estimates of SARS-CoV-2 transmission rates: Associations between mobile telephone data tracking and R. J. Public Health Manag. Pract..

[15] Jang S, Han SH, Rhee JY (2020). Cluster of coronavirus disease associated with fitness dance classes,South Korea. Emerg. Infect. Dis..

[16] Waltenburg MA (2020). Update: COVID-19 among workers in meat and poultry processing facilities—United States, April–May 2020. MMWR Morb. Mortal Wkly. Rep..

[17] Burak KW (2021). COVID-19 outbreak among physicians at a Canadian curling bonspiel: A descriptive observational study. CMAJ Open.

[18] Cauh NVC (2021). Superspreading event of SARS-CoV-2 infection at a bar, Ho Chi Minh City, Vietnam. Emerg. Infect. Dis..

[19] Qian H (2020). Indoor transmission of SARS-CoV-2. Indoor Air.

[20] Jones B (2021). SARS-CoV-2 transmission during rugby league matches: do players become infected after participating with SARS-CoV-2 positive players?. Br. J. Sports Med..

[21] Johansson MA (2021). SARS-CoV-2 transmission from people without COVID-19 symptoms. JAMA Netw. Open.

[22] Hacisuleyman E (2021). Vaccine breakthrough infections with SARS-CoV-2 variants. N. Engl. J. Med..

[23] Lange B, Gerigk M, Tenenbaum T (2021). Breakthrough infections in BNT162b2-vaccinated health care workers. N. Engl. J. Med..

[24] Moreno GK (2021). SARS-CoV-2 transmission in intercollegiate athletics not fully mitigated with daily antigen testing. Clin. Infect. Dis..

